# A multifunctional therapeutic approach to disease modification in multiple familial mouse models and a novel sporadic model of Alzheimer’s disease

**DOI:** 10.1186/s13024-016-0103-6

**Published:** 2016-04-29

**Authors:** Jia Luo, Sue H. Lee, Lawren VandeVrede, Zhihui Qin, Manel Ben Aissa, John Larson, Andrew F. Teich, Ottavio Arancio, Yohan D’Souza, Ahmed Elharram, Kevin Koster, Leon M. Tai, Mary Jo LaDu, Brian M. Bennett, Gregory R. J. Thatcher

**Affiliations:** Department of Medicinal Chemistry and Pharmacognosy, College of Pharmacy, University of Illinois at Chicago, Chicago, IL USA; Department of Psychiatry, Neuropsychiatric Institute, University of Illinois at Chicago, Chicago, IL USA; Department of Pathology, The Taub Institute for Research on Alzheimer’s Disease and the Aging Brain, Columbia University, New York, NY USA; Department of Biomedical & Molecular Sciences, Faculty of Health Sciences, Queen’s University, Kingston, ON Canada; Department of Anatomy and Cell Biology, College of Medicine, University of Illinois at Chicago, Chicago, IL USA

**Keywords:** CREB activation, NO/cGMP signaling, CMZ, NMZ, Neuroprotection, Alzheimer’s disease modifying approach, Mixed pathology dementia, AD mouse models, *Aldh2*^*−/−*^ mice

## Abstract

**Background:**

Clinical failures singularly targeting amyloid-β pathology indicate a critical need for alternative Alzheimer’s disease (AD) therapeutic strategies. The mixed pathology reported in a large population of AD patients demands a multifunctional drug approach. Since activation of cAMP response element binding protein (CREB) plays a crucial role in synaptic strengthening and memory formation, we retooled a clinical drug with known neuroprotective and anti-inflammatory activity to activate CREB, and validated this novel multifunctional drug, NMZ, in 4 different mouse models of AD.

**Results:**

NMZ was tested in three mouse models of familial AD and one model of sporadic AD. In 3 × Tg hippocampal slices, NMZ restored LTP. In vivo, memory was improved with NMZ in all animal models with robust cognitive deficits. NMZ treatment lowered neurotoxic forms of Aβ in both APP/PS1 and 3 × Tg transgenic mice while also restoring neuronal plasticity biomarkers in the 3 × Tg mice. In EFAD mice, incorporation of the major genetic AD risk factor, h*APOE4*, did not mute the beneficial drug effects. In a novel sporadic mouse model that manifests AD-like pathology caused by accelerated oxidative stress in the absence of any familial AD mutation, oral administration of NMZ attenuated hallmark AD pathology and restored biomarkers of synaptic and neuronal function.

**Conclusions:**

The multifunctional approach, embodied by NMZ, was successful in mouse models of AD incorporating Aβ pathology (APP/PS1), tau pathology (3xTg), and *APOE4,* the major human genetic risk factor for AD (EFAD). The efficacy observed in a novel model of sporadic AD (*Aldh2*^*−/−*^) demonstrates that the therapeutic approach is not limited to rare, familial AD genetic mutations. The multifunctional drug, NMZ, was not designed directly to target Aβ and tau pathology; however, the attenuation of this hallmark pathology suggests the approach to be a highly promising, disease-modifying strategy for AD and mixed pathology dementia.

## Background

Alzheimer disease (AD), the most common form of dementia, has reached epidemic proportions, presenting a severe economic and social burden worldwide. Currently, no effective therapy exists. Early onset familial AD (FAD) which represents 1-4 % of cases [1], is linked to mutations in *APP*, *PS1*, and *PS2* which increase deposition of insoluble amyloid-β peptide (Aβ) [2]. According to the Amyloid Hypothesis, Aβ leads to neuronal death and the second hallmark neuropathology, tau-containing neurofibrillary tangles (NFT). Consequently, the search for AD therapeutics has been dominated by use of transgenic mouse models that overexpress mutant human genes linked to familial AD. Of AD cases, >96 % are multifactorial in origin, and importantly, hallmark AD pathology is often accompanied by other neuropathologic changes. Not unexpectedly, in this substantial population with AD neuropathology, the correlation of Aβ histopathology with cognitive decline is poor [3, 4]; providing one possible contributor to the failure of at least eight therapeutics in late stage clinical trials singularly targeting Aβ [5, 6]. While not dismissing a role for Aβ in AD pathogenesis, cogent arguments have been made that a new strategy is needed towards an effective pharmacotherapeutic response [7–10].

**Fig. 1:**
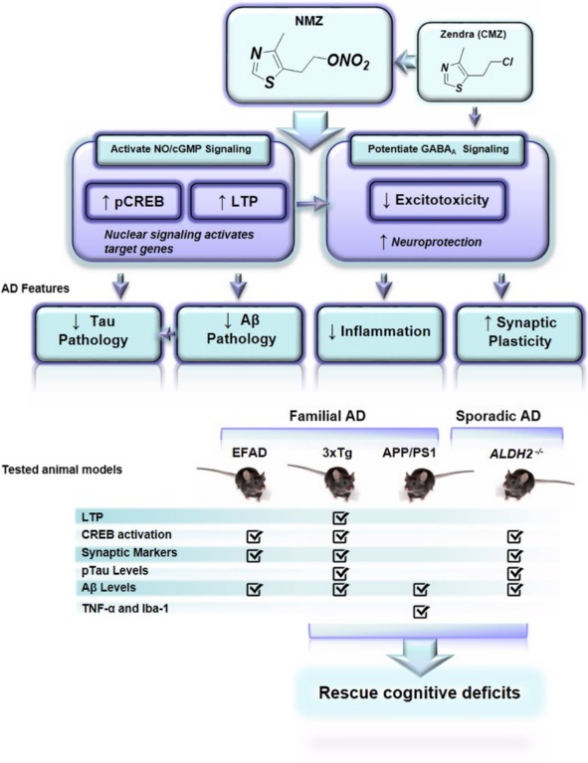
Redesigning of the anticonvulsant Zendra for treatment of AD and dementia. The redesigned small molecule, NMZ, should retain the reported neuroprotective properties, while additionally targeting synaptic dysfunction, the case for which in AD is persuasive [16]. NMZ was designed to restore synaptic function via NO/cGMP/CREB signaling, and to retain the beneficial actions of CMZ. The desired activity profile was observed in multiple animal models on treatment with NMZ. Table indicates a summary of performed assays in used mouse models

Expert opinion has recognized that AD treatment must target two or more factors in disease pathogenesis [8]. Drug repositioning is also attractive with particular promise shown by anticonvulsants [11]. Zendra (chlomethiazole, CMZ), an anticonvulsant with a history of clinical use, has been shown extensively to be neuroprotective against oxidative stress in animal models and targets inflammation, mitochondrial dysfunction, and excitotoxicity [12–14]; all of which are proposed to contribute to AD pathogenesis. CMZ is suggested as a potential component of future combination therapies for neuronal injury [14], and we have shown that CMZ protected primary neurons against oxygen glucose deprivation (OGD) and neurotoxic oligomeric Aβ (oAβ) insult [15]. Additional contributors to onset and progression of AD include early synaptic failure [16], cerebrovascular impairment [17], and depletion of neurotrophins [18]. We hypothesized that a small chemical modification to CMZ, as in the novel therapeutic agent, 4-methyl-5-(2-(nitrooxy) ethyl) thiazol-3-ium chloride (NMZ), would address these contributors (Fig. 1) [19, 20].

Our approach was inspired by the early work of Lipton [21] and that of Kandel linking nitric oxide (NO) and cyclic guanosine monophosphate (cGMP) to cAMP-response element binding protein (CREB) activation and long-term potentiation (LTP) [22]. Activation of CREB by phosphorylation has been shown to be necessary for memory formation and synaptic strengthening, therefore we hypothesized that the incorporation of a NO/cGMP signaling capability in NMZ would restore circuits dependent on CREB activation essential for learning and memory [23] (Fig. 1). Recent evidence links decreased CREB activity and dysregulated CREB-mediated gene regulation directly to the pathogenesis of AD [24–26]. Therefore NMZ, although not targeted at a specific component of Aβ accumulation, might reduce brain Aβ consequent to activation of NO/cGMP/CREB [27, 28]. We previously demonstrated that NMZ retained attributes of CMZ including potentiation of gamma-aminobutyric acid (GABA) signaling at the GABA_A_ receptor [19, 20]. We also reported procognitive actions associated with NO/cGMP, including reversal of scopolamine-induced cognitive deficits, and demonstrated a high therapeutic index and efficacy at low nanomolar concentrations of NMZ in the brain [19].

Testing of NMZ was initiated in a variety of models demonstrating various subtypes of AD pathology, achieving a task set by expert opinion, in engagement of multiple targets in multiple AD transgenic models [8, 29]. First, NMZ was tested in the well-established young APP/PS1 and older 3 × Tg transgenic mouse models of FAD. LTP in hippocampal slices was restored by NMZ, in both APP/PS1 and 3 × Tg mice. Since human *apolipoprotein E4* (*APOE4*) [30] is the strongest genetic risk factor for sporadic AD, testing proceeded to the E4FAD AD mouse model that incorporates targeted replacement of mouse *APOE* with h*APOE4.* Oxidative stress is a primary driving force in AD pathogenesis [31, 32], linked to the lipid peroxidation product 4-hydroxynonenal (HNE) that accumulates in the brains of late-stage and presymptomatic AD patients, and is linked to apoE4 negative function [33, 34]. Loss of the enzyme aldehyde dehydrogenase 2 (ALDH2), which is important for 4-hydroxynonenal (4-HNE) detoxification, leads to HNE accumulation, neurodegeneration, and memory loss [35]*.* Therefore, NMZ was tested in the *Aldh2*^*−/−*^ mouse that develops age-dependent cognitive deficits [36], providing validation of NMZ in a model of sporadic AD. NMZ, as a brain-bioavailable small molecule, was observed to protect against neuronal loss and synaptic dysfunction, improve memory, lower pro-inflammatory cytokines, and attenuate hallmark Alzheimer’s pathology in these multiple mouse models.

## Results

### NMZ protects primary neurons from oAβ and oxygen glucose deprivation insult

**Fig. 2:**
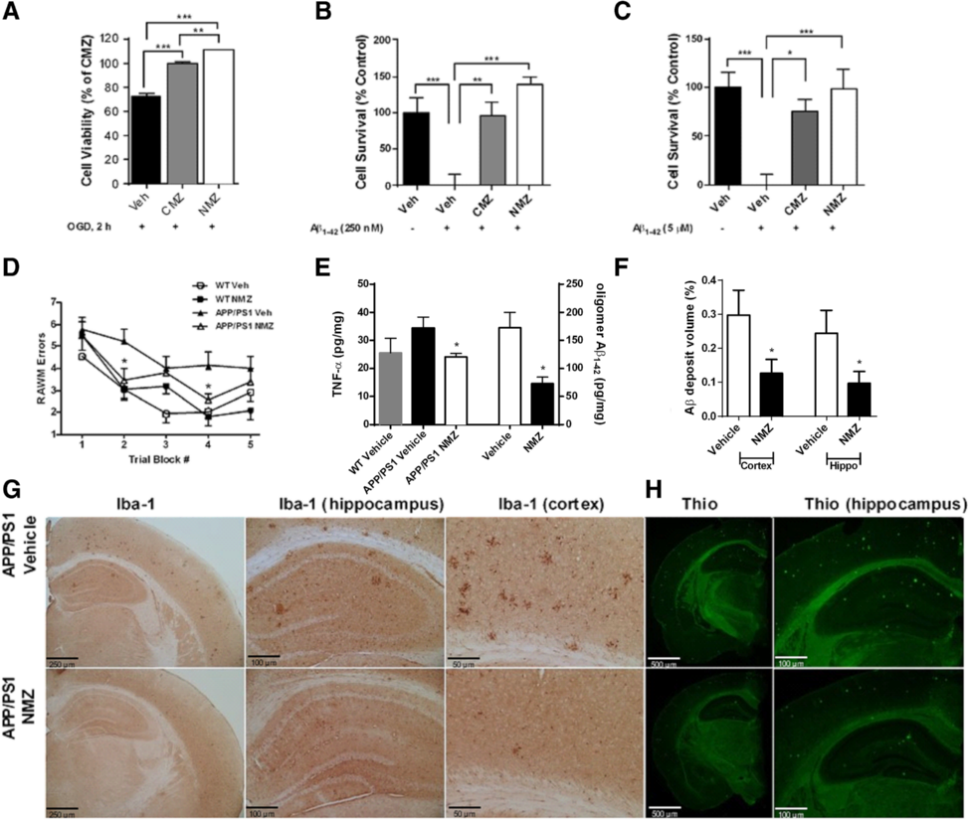
Retention of neuroprotective activity (**a**-**c**) and the effects of NMZ treatment in APP/PS1 mice (**d**-**h**). Primary neuronal cultures treated with NMZ showed neuroprotection when exposed to 2 h OGD (**a**) and both low (**b**) and high (**c**) doses of Aβ oligomers. Data show mean ± S.E.M. normalized to insult and non-insult vehicle controls. Male APP/PS1 mice (n = 6–8) treated for 12 weeks with NMZ (1 mg/kg, i.p. plus 20 mg/kg/day in drinking water) demonstrated an improvement in the RAWM task (**d**) and reduction in levels of the pro-inflammatory marker, TNF-α, and in neurotoxic oAβ_,_ in whole brain homogenates (**e**). Data show mean ± S.E.M compared to vehicle. NMZ also reduced total Aβ_1–42_ deposit volumes compared to vehicle control in cortex and hippocampal homogenates (**f**). Data show mean ± S.E.M of total deposit volume determined by fluorescent staining and were analyzed by two-tailed student’s *t*-test. All statistical significance is indicated by *p < 0.05, **p < 0.01, ***p < 0.001. Representative images of hippocampi and cortices with histochemical staining reveal significantly less activated microglia shown with Iba-1 (magnification: 4×, 10×, and 20× from left to right) (**g**) and reduced thioflavin-S staining of amyloid plaques in NMZ treatment compared to vehicle (magnification: 2× and 6×) (**h**)

NMZ retained the neuroprotective actions of CMZ [15], providing efficacy against OGD (Fig. 2a) and against exposure to oAβ (250nM or 5 μM). Cell viability was reduced by ~ 15 % and 60 % after vehicle treatment and exposure to oAβ, 250nM and 5 μM, respectively (Fig. 2b, c). NMZ demonstrated superior protection compared to CMZ in mitigating oxidative and low and high dose oAβ insults.

### In APP/PS1 mice, NMZ restores cognition and lowers Aβ

NMZ was first tested in APP/PS1 double transgenic mice that manifest aberrant accumulation of human Aβ driven by FAD mutations in APP_695_^K670N/M671L^and PS1^M146L^. We have previously measured NMZ brain bioavailability and correlated brain levels with efficacy in cognition enhancement in scopolamine-induced amnestic mice, demonstrating that the brain concentration of NMZ required for memory consolidation after amnestic insult is approximately 0.5–1.0 nM [19]. Our previous PK/PD studies [19] guided dose selection herein, since NMZ was effective by both i.p. and p.o. drug administration at doses of 1 mg/kg and 20 mg/kg/day, respectively. Therefore, NMZ was administered at 1 mg/kg/day i.p. and 20 mg/kg/day in drinking water, treating randomized groups of both transgenic and age- and sex-matched wildtype (WT) littermates from 2.5 months of age for 12 weeks. In the last 2 weeks of treatment, animals were tested in the radial arm water maze (RAWM). NMZ treated transgenic animals performed significantly better than APP/PS1 control animals in trial blocks 2 and 4 (*p* < 0.05) (Fig. 2d).

In addition to improvement of cognition, whole brain homogenates were analyzed for various biomarkers. In whole brain homogenates, a significant reduction of oAβ was observed for NMZ treated animals for Aβ_1–42_ species (Fig. 2e). Classic Aβ pathology was studied using quantitative Thioflavin S stereological analysis of serial coronal sections. NMZ treatment demonstrated almost a 3-fold reduction in Thioflavin S-positive Aβ deposit volume in both the cortex and hippocampus (Fig. 2f and h). Lower levels of inflammatory markers in human blood and in rat models of hypoxia-ischemia in vivo and ex vivo, including the pro-inflammatory cytokine tumor necrosis factor alpha (TNF-α), have been reported previously for CMZ [12]. Total brain homogenates from APP/PS1 mice and WT littermates analyzed for TNF-α showed significant NMZ-induced reduction (Fig. 2e). This was mirrored by Iba-1 staining of coronal slices that showed reductions in activated microglia in the hippocampi and cortex of NMZ treated mice (Fig. 2g). Thus, in the one FAD model, in which WT mice are littermates, NMZ treatment significantly reduced elevation of TNF-α; a result compatible with the reported anti-inflammatory actions of CMZ.

### In 3xTg mice, NMZ restores LTP via NO/cGMP, reverses cognitive deficits, and reduces Aβ and p-tau

**Fig. 3:**
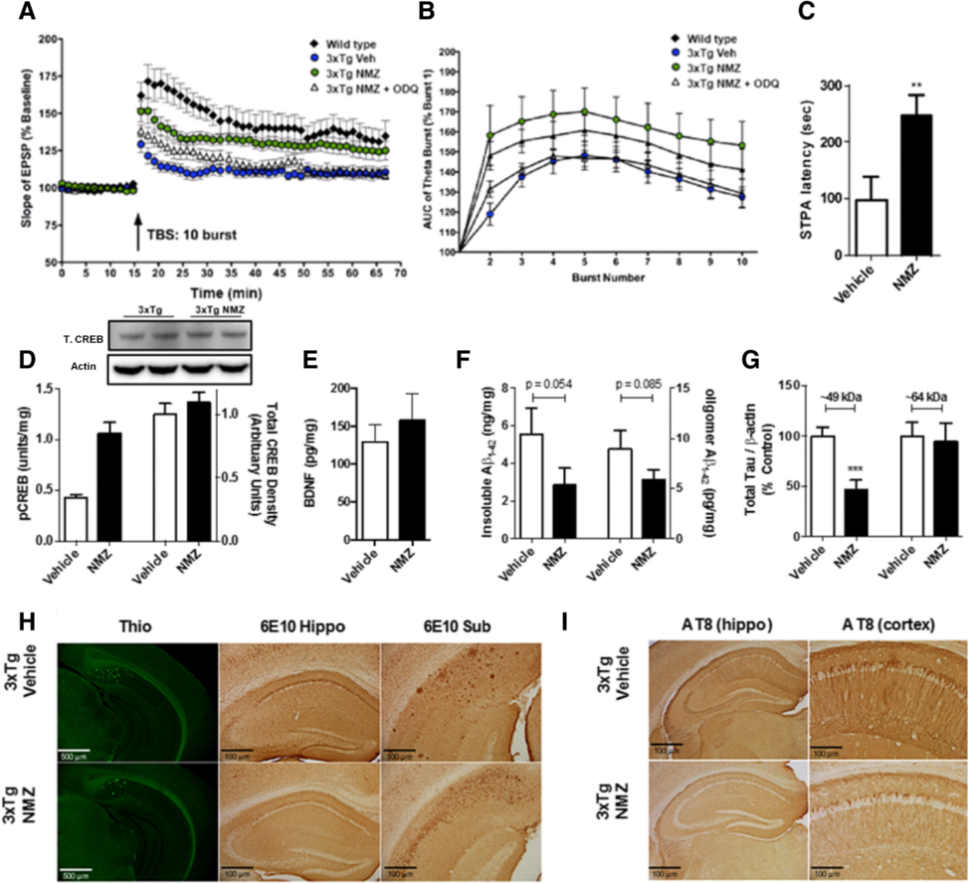
NMZ treated 3xTg mice show improvements in LTP, behavior, synaptic biomarkers, and Aβ pathology. LTP was measured in the CA1 region of hippocampal sections from 16 month male 3 × Tg mice (*n* = 9-12) or in littermate controls treated with NMZ (50 μM) and ODQ (10 μM) 30 min prior to TBS and continued throughout. Fifty min after LTP induction, NMZ showed restoration of LTP deficits to WT levels, while ODQ completely blocked this activity (**a**). Analysis of theta bursts showed an increase by NMZ during induction of long-term potentiation (**b**). Data show mean ± S.E.M. normalized to baseline. Male and female 3xTg mice (*n* = 8–12) treated for 10 weeks with NMZ (1 mg/kg, i.p. + 20 mg/kg/day p.o) showed reversal of memory deficits to levels observed in WT mice in the STPA task 24 h after training (**c**). NMZ treatment resulted in an elevation of biomarkers associated with synaptic and neuronal plasticity (pCREB and BDNF) in whole brain homogenates measured by ELISA however not changing the total amount of total CREB measured by western blot (**d** and **e**). NMZ treatment attenuated insoluble and oligomeric Aβ (**f**) and total tau (**g**), both markers of AD hallmark pathology, in whole brain homogenates. Data show mean ± S.E.M. compared to vehicle control. Statistical significance is indicated by *p < 0.05, **p < 0.01, ***p < 0.001 and were analyzed by unpaired student’s *t*-test. NMZ treatment showed a decrease in Aβ staining in the CA1 region of the hippocampus in treated animals but a negligible effect on the cortex. Representative images are shown with thioflavin-S (magnification 4×) staining and 6E10 antibody in the hippocampus (magnification 4×) and subiculum (magnification 10×) (**h**). AT8 immunohistochemistry in hippocampus and cortex (magnification 6× and 20×) showed a decrease in phosphorylated tau in the NMZ treated compared to the vehicle (**i**)

The increased age of LaFerla’s 3 × Tg (APP_695_^K670N/M671L^, PS1^M146L^, tau^P301L^) transgenic mouse model at treatment onset and the more complex pathology represents an increased therapeutic challenge. The effect of NMZ was first studied in LTP in the CA3-CA1 pathway in hippocampal slices from 16-month-old male mice [37]. Since the 3 × Tg is a homozygous line, the 129/SvJ x C57BL/6 background was used as an outbred line to provide control slices to give baseline performance. NMZ treatment resulted in a significant increase in fEPSP after LTP induction compared to untreated transgenics, to levels similar to WT control (Fig. 3a). To investigate the involvement of the NO/cGMP pathway in the action of NMZ, ODQ (1H-[1, 2, 4] oxadiazolo-[4, 3-a] quinoxalin-1-one), a selective inhibitor of soluble guanylyl cyclase (sGC) was used to block cGMP production, and added 30 min prior to induction. LTP induced in the presence of both NMZ and ODQ was not significantly different to that obtained in untreated 3 × Tg slices. Examination of the field responses during theta-burst stimulation (TBS) indicated that NMZ facilitated LTP in 3 × Tg mice by enhancing the depolarization induced by the high frequency bursts (Fig. 3b); this effect was also attenuated by inhibition of cGMP production by ODQ.

In light of the findings in APP/PS1 mice, we chose to use a similar treatment protocol in 3 × Tg mice. Drug treatment started at 10 months of age for females and 12 months of age for males (to account for early pathology in females). In the last week of the trial, the animals were tested in the step through passive avoidance (STPA) task. The performance of NMZ treated 3 × Tg mice was comparable to untreated WT and significantly better than vehicle treated 3xTg mice (Fig. 3c). The procognitive activity of NMZ is proposed to be mediated via re-activation of CREB, leading to increased expression of gene products, notably brain-derived neurotrophic factor (BDNF). In cerebral homogenates, both pCREB and BDNF levels were significantly increased in NMZ treated 3 × Tg mice (Fig. 3d and e). Aβ_1–42_ measured in the insoluble guanidine extracted fraction and oAβ in the soluble fraction was lowered with NMZ treatment (Fig. 3f). Staining using the Aβ antibody 6E10 revealed fewer β-amyloid plaques in the hippocampal formation, particularly in the subiculum (Fig. 3h), although the cortex did not show a significant difference in plaque pathology. Immunohistochemical analysis using AT8 an antibody directed against phosphorylated tau showed a significant decrease in staining in the CA1 region of NMZ treated mouse brains (Fig. 3i). In contrast, total tau was unchanged, measured by western blot of total brain extract using antibody HT7 (a human-specific monoclonal antibody recognizing amino acids 159–163) (Fig. 3g).

### In EFAD mice, NMZ lowers Aβ and elevates CREB phosphorylation and PSD-95

**Fig. 4:**
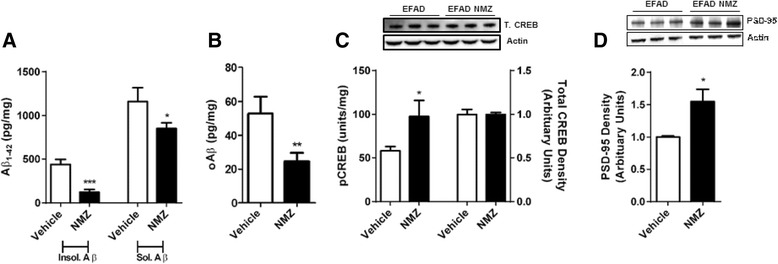
NMZ treated E4FAD mice show improvements in Aβ pathology and synaptic biomarkers. Male E4FAD (*n* = 8–12) mice treated with NMZ for 12 weeks (1 mg/kg, i.p. + 20 mg/kg/day p.o.) showed attenuated formation of insoluble and soluble Aβ_1–42_ (**a**) and oligomeric Aβ (**b**) in hippocampal homogenates measured by ELISA. NMZ treatment led to elevated pCREB in the hippocampal fraction (**c**) without a change in total CREB determined by western blot. NMZ treated mice restored levels of the synaptic marker PSD-95 determined by western blot (**d**). Data show mean ± S.E.M. compared to the vehicle control. Statistical significance is indicated by *p < 0.05, **p < 0.01, ***p < 0.001 and were analyzed by unpaired student’s *t*-test

E4FAD mice carry targeted replacement of mouse *APOE* with human *APOE4*. The 5 × FAD background causes deposition of human Aβ, which is more severe in apoE4 carrier mice than in mice carrying the apoE2 or apoE3 isoforms [30]. The E4FAD mouse is therefore an important AD model for drug testing of predictive efficacy in the human apoE4 carrier population. Male E4FAD mice at 3.5 months of age were, as in the previous mouse models, administered NMZ i.p. (1 mg/kg/day) and p.o. in hydrogel (20 mg/kg/day) for 12 weeks before sacrifice, allowing comparison with published studies on male EFAD mice at the same age [38]. A significant cognitive deficit was not observed using the STPA task, nor nesting behavior assessment up to 6 months of age, compared to C57BL/6 WT mice. However, after tissue extraction, Aβ_1–42_ showed a significant reduction in the soluble, insoluble and oligomeric forms (Fig. 4a, b). Levels of pCREB and total CREB, measured in hippocampal homogenates, showed a significant increase in pCREB and no significant differences in total CREB in the NMZ treatment group (Fig. 4c). Activation of CREB is intrinsically linked to synaptic function; and the scaffold protein, postsynaptic density protein 95 (PSD-95), plays a critical role in synaptic plasticity and synaptic dysfunction in AD [39, 40]. Immunoassay of homogenates from E4FAD mice showed a significant increase of PSD-95 in the hippocampus of E4FAD mic3 after treatment with NMZ (Fig. 4d).

### In Aldh2 ^−/−^ mice, NMZ restores synaptic plasticity and attenuates AD hallmark pathology

**Fig. 5:**
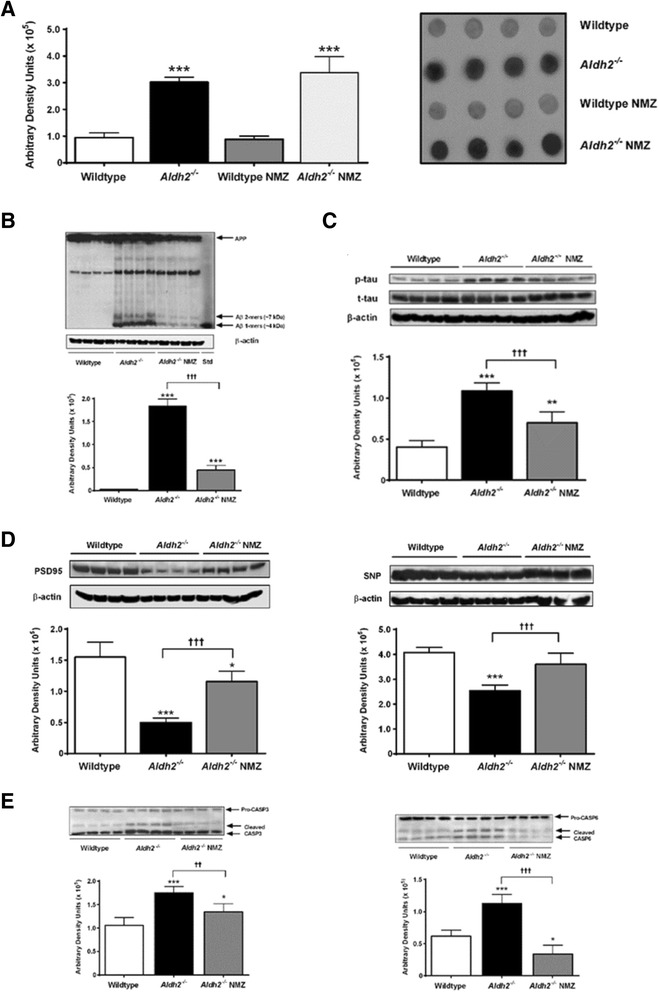
NMZ treated Aldh2^−/−^ mice show improvements in AD-like pathology, synaptic biomarkers and neuronal function. Hippocampal homogenates (30 μg protein) from *Aldh2* ^*−/−*^ mice, treated with NMZ or vehicle for 12 weeks, showed no effect of NMZ on HNE adduct formation by dot-blot immunoassay of HNE protein adducts; whereas HNE adducts were significantly elevated relative to treated and untreated WT mice mice (**a**). Immunoblot analysis of hippocampal homogenates of WT and drug or vehicle treated *Aldh2* ^−/−^ mice quantitated by densitometry with representative blots shown: monomeric Aβ (**b**) and p-tau (**c**); synaptic biomarkers PSD-95 and synaptophysin (**d**); and activated caspases (**e**). Data are presented as the mean ± S.D. and were analyzed by one-way ANOVA with Bonferroni post-hoc tests. Statistical significance is indicated by *p < 0.05, **p < 0.01, ***p < 0.001 compared with wildtype; †, significant difference from *Aldh2* ^−/−^ (†† *p* < 0.01, ††† *p* < 0.001)

*Aldh2*^−/−^ mice, compared to age and sex-matched littermates, show a progressive decrease in performance, beginning at 2.5–3 months of age, in both the novel object recognition (NOR) and Y-maze tasks, measures of non-spatial and spatial working memory [36]. Given the positive outcomes of NMZ treatment in the three familial AD transgenic mouse models, we decided to forego i.p. delivery in *Aldh2*^−/−^ mice, administering drug solely by the oral route (20 mg/kg/day) for 12 weeks from 3 months of age. The *Aldh2*^−/−^ mouse represents a model for accelerated aging manifested by accumulation of lipid peroxidation products that fail to be cleared by Aldh2 enzyme action and pathology reflected by significantly increased levels of HNE-adducted proteins. We hypothesized that NMZ would counteract the downstream effects of this pathology on neuronal and synaptic function without impacting upstream HNE-adduct formation. Marked increases in HNE adduct formation occur early in *Aldh2*^−/−^ mouse brains, characterized by increases in both the density and number of immunoreactive bands in immunoblots, which was not significantly perturbed by administration of NMZ (Fig. 5a). *Aldh2*^−/−^ mice demonstrated age-related increases in soluble Aβ and phospho-tau that were significant at 6 months of age [36] and attenuated by NMZ treatment (Fig. 5b, c), without change in total tau. Age-related changes in synaptic and neuronal markers were also significantly attenuated by NMZ treatment (Fig. 5d), as were age-related increases in activated caspases 3 and 6 (Fig. 5e). These progressive biochemical changes linked to synaptic and neuronal dysfunction are intrinsically linked with AD in humans.

**Fig. 6:**
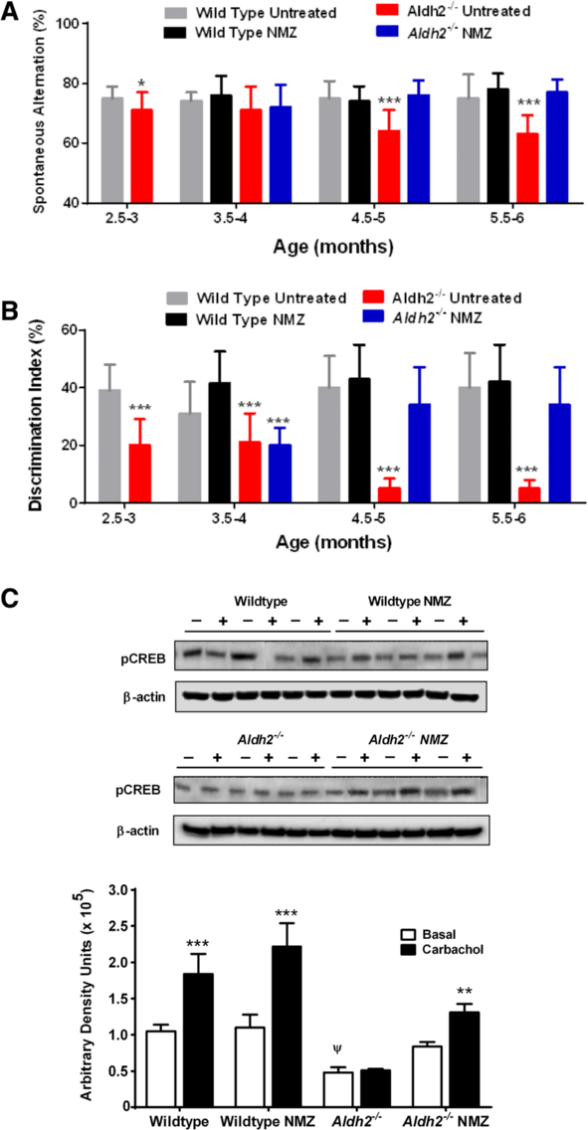
NMZ-treated Aldh2^−/−^ mice show rescued learning, memory and CREB responsiveness in carbachol treated hippocampal slices. Reversal of the age-dependent decline in the spontaneous alternation rate and discrimination index in the Y-maze task (**a**) and NOR task (**b**), respectively, was observed in male and female *Aldh2* ^*−/−*^ mice: after obtaining baseline measurements at 2.5–3 months, mice were randomized to drug or vehicle control groups (*n* = 8–11) and treated with NMZ (20/mg/kg/day p.o.) or vehicle at 3 months of age for a period of 12 weeks. Pre-randomization data were compared by an unpaired *t*-test and post-randomization groups by a one-way ANOVA with a Bonferroni post-hoc test. Hippocampal slices from 6 month old wild type and *Aldh2* ^*−/−*^ mice that had been treated with NMZ or vehicle control for 12 weeks, were incubated with 50 μM carbachol or vehicle (Basal) for 30 mins and snap frozen. Immunoblot analysis for pCREB was performed using 30 μg protein of hippocampal homogenate, and immunoreactive bands were quantitated by densitometry (**c**). Data are presented as the mean ± S.D. (*n* = 3) and were analyzed by a one-way ANOVA with a Bonferroni post-hoc test: * significant differences from basal (** *p < 0.01,* ***p < 0.001); ψ significant difference compared to basal in all other groups (*p* < 0.05)

*Aldh2*^−/−^ and WT mice were tested in the Y-maze and NOR before randomization into treatment groups. In both tasks, untreated *Aldh2*^−/−^ mice showed a significant cognitive deficit that was more distinct with age; NMZ treatment having no effect on WT mice. A significant deficit was seen in *Aldh2*^−/−^ mice prior to randomization; whereas, NMZ treated mice up to 6 months of age showed no cognitive deficit in the Y-maze and NOR (Fig. 6a, b).

A final ex vivo experiment was carried out using the acetylcholine receptor agonist, carbachol, applied to hippocampal slices from NMZ-treated *Aldh2*^−/−^ mice, WT littermates, and their vehicle controls. Carbachol stimulation of hippocampal slices from 6 month old WT mice that had been administered NMZ or vehicle control for 12 weeks caused significant and similar elevation of pCREB (Fig. 6c). Slices from vehicle treated *Aldh2*^*−/−*^ mice showed significantly attenuated basal pCREB response relative to WT and no significant effect of carbachol stimulation, whereas hippocampi from *Aldh2*^*−/−*^ mice that had undergone NMZ treatment showed basal response and stimulation in response to carbachol comparable to WT littermates. NMZ treatment of *Aldh2*^*−/−*^ mice was able to restore normal basal and stimulated pCREB signaling.

## Discussion

The collected data presented herein profile an orally bioavailable small molecule, NMZ, that yields beneficial biochemical and functional outcomes in four diverse mouse models of Alzheimer’s disease, building on animal data showing the ability of NMZ to restore memory consolidation in response to a variety of acute amnestic insults and in response to saporin-induced cholinergic lesions [19, 41]. Lowering brain Aβ levels has remained the primary and often exclusive target for therapeutic intervention in AD; however, the failure of such therapeutic agents to reach primary endpoints in phase 3 clinical trials, emphasizes the urgent need for new approaches. Nevertheless, any new therapeutic approach to AD should also positively impact hallmark neuropathology. NMZ targets multiple contributing mechanisms in AD and dementia. Beginning with the APP/PS1 mouse that manifests Aβ pathology and cognitive deficits, the efficacy of this approach was demonstrated. Adding tau pathology in older 3 × Tg mice and h*APOE4* in E4FAD mice did not blunt the efficacy of NMZ, which was also demonstrated in a novel mouse model of sporadic AD.

AD accounts for up to 80 % of dementia and half of those diagnosed with dementia have more than one pathology; for example vascular dementia, and dementia with Lewy bodies. However, in the population with AD pathology, Aβ histopathology with cognitive deficits have shown to be poorly correlated [3, 4], and Aβ-specific therapeutics might be expected to fail in this clinical population. AD is a multifactorial disease linked to other aging-associated maladies and to factors common to other neurodegenerative diseases, for example, glutamate excitotoxicity, oxidative stress, and inflammation [42]. Neuroprotective agents have been targeted to these and other contributors to neurodegeneration. For example, the clinical anticonvulsant Zendra/CMZ, was repurposed in Phase 3 clinical trials as a neuroprotective drug for use in spinal cord injury and ischemic stroke. CMZ, clinically prescribed for anxiety and agitation in the elderly, continues to be recommended as a component of combination therapy for stroke and neuronal injury [14]. GABA-potentiation [43] and TNF-α antagonism [44, 45] have been targeted for AD therapy and there is evidence that CMZ provides these attributes in addition to attenuation of mitochondrial and neuronal damage [12]. The early loss of synaptic function in both AD and mild cognitive impairment (MCI), which correlates with cognitive dysfunction and memory loss [46, 47], must be addressed; therefore CMZ, was chemically modified in the form of NMZ, to treat Alzheimer’s and dementia [20] (Fig. 1). Importantly, NMZ was shown to retain the beneficial properties of CMZ [19].

Studies suggest that restoration of synaptic failure in AD can be achieved through CREB activation [48], and since CREB activation is coupled to NO/cGMP signaling in the hippocampus [22, 23], we hypothesized that incorporation of a CREB activating moiety would reactivate circuits associated with CREB which are vital to learning and memory [49]. NMZ was designed to activate CREB through the NO/cGMP signaling pathway [19], regulating the strength of synaptic transmission in an activity-dependent manner in the hippocampus [50] (Fig. 1). NMZ was effective in 3 × Tg mice, wherein LTP restoration was shown to be cGMP dependent (Fig. 3).

NMZ significantly restored cognitive function in different memory tasks in 6 month old APP/PS1 and 12–15 month old 3 × Tg mice (Figs. 2 and 3). Given this positive response in FAD mice to NMZ administered using daily i.p. delivery supplemented by oral delivery over 12 weeks, administration of NMZ to *Aldh2*^−/−^ mice was reduced to oral delivery alone. Working memory was measured in two further memory tasks (Fig. 6), showing a progressive decline in performance in *Aldh2*^−/−^ mice compared to the WT littermate control group. Before randomization for drug treatment at 2.5–3 months, *Aldh2*^−/−^ mice showed a deficit in both tasks. After 4 weeks treatment with NMZ, spontaneous alternation in the Y-maze was not different from that of WT controls; and after 8 weeks treatment, NOR performance recovered to that of WT controls. Thus, in 4 different behavioral assays of working and spatial memory and 3 different animal models, NMZ administration rescued the age-related decline in learning and memory.

Molecular network analysis and other clinical evidence converges on aberrant CREB-mediated gene regulation in the AD hippocampus [25, 26], compatible with the role of CREB phosphorylation in mediating synaptic plasticity through structural changes at synapses. In NMZ-treated 3×Tg, E4FAD, and *Aldh2*^−/−^ mice, levels of pCREB in either whole brain homogenates or hippocampi were significantly increased over vehicle control (Figs. 2, 3, 4 and 5). CREB acts upon downstream genes involved in synaptic and neuronal plasticity and neurogenesis, including BDNF. NMZ caused elevation of BDNF and of the protein biomarker of synaptic function, PSD-95 (Figs. 3, 4 and 5). The inability of CREB, in hippocampal slices from *Aldh2*^−/−^ mice, to respond to cholinergic stimulation further mirrors the loss of function of CREB in AD (Fig. 6). Given the evidence for direct dysregulation of CREB by oAβ and reciprocal regulation of amyloidogenesis by CREB [27, 28, 51], an agent that elevates pCREB and biomarkers of synaptic function in the AD brain may have a reciprocal and beneficial effect on production and/or clearance of Aβ. More direct links between NO/cGMP signaling and APP processing via beta-secretase-1 (BACE1) have been reported: the anti-amyloidogenic activity of an NO-donor was reported to be BACE1 and cGMP-dependent [52, 53], and intact endothelial NO synthase (eNOS) activity in the cerebral microvasculature [54] and brain tissues is linked with downregulation of BACE1 activity [52, 55, 56]. In three models of FAD, treatment with NMZ resulted in attenuated levels of Aβ (Figs. 2, 3, 4, 5 and 6).

Contemporary disease-modifying approaches to AD remain targeted at lowering brain Aβ aggregates, including immune-based approaches. Most recently the gantenerumab Aβ antibody was shown preferentially to bind aggregated brain Aβ and in APP/PS-1 mice, to reduce or halt Aβ plaque formation [57]; however, the Phase 3 clinical trial, in early stage symptomatic patients with evidence of amyloid, was halted in December 2014. Since AD is defined by the hallmark neuropathologies of aberrant Aβ and tau protein aggregates, any new disease-modifying therapy should attenuate these pathologies. The data presented herein for NMZ in 4 preclinical animal models suggest that therapeutic strategies targeting synaptic and neuronal dysfunction are also able to ameliorate hallmark pathology. NMZ treatment was shown to lower p-tau in the 3 × Tg mouse using antibodies that selectively recognize neurofibrillary filaments made up of hyperphosphorylated human tau and also to attenuate native p-tau formation in the *Aldh2*^−/−^ mouse (Figs. 3 and 5).

## Conclusions

Disease-modifying approaches to AD remain predominantly targeted at lowering Aβ aggregates in the brain. The development of effective therapies for AD and mixed pathology dementia has been limited by the lack of suitable animal models adequately reflecting the complexity of human AD. We successfully modified a neuroprotective, anti-inflammatory drug to retain these functions and to address synaptic dysfunction in AD: the new orally bioavailable small molecule was effective in three FAD models of increasing therapeutic challenge and in a new animal model of AD not linked to rare genetic mutations. The multifunctional approach incorporates NO/cGMP signaling which regulates the strength of synaptic transmission in an activity-dependent manner in the hippocampus [50]. Importantly, NMZ reactivated CREB signaling, effectively restored LTP, improved learning and memory, and attenuated TNFα and pathological hallmarks of AD. These observations should encourage alternate, parallel strategies to the singular targeting of Aβ. The increasing recognition that AD and age-related dementia may have underlying mixed pathology further supports a small molecule multifunctional approach to neurodegeneration and dementia.

## Methods

### Animals

All animal care and procedures were conducted with approved institutional animal care protocols at UIC (OACIB), Columbia University (IACUC) and Queen’s University (UACC), in accordance with the NIH Guide for the Care and use of Laboratory Animals.

Mice were randomly grouped into NMZ or vehicle treatment groups and mice were treated with drug or vehicle for 10–12 weeks; where applicable littermates were sex and age-matched. APP/PS1 mice (6–8 weeks) were administered drug (1 mg/kg qd) or saline i.p. supplemented with drug (20 mg/kg qd in drinking water). 3 × Tg mice (males 12 months; females 10 months) and E4FAD mice (male 4 months) were administered drug using the same protocol, with the exception that drug in hydrogel replaced drinking water [38]. *Aldh2*^*−/−*^ mice (3 months) were treated with drug (20 mg/kg qd) in hydrogel. Primary cultures of cortical neurons were prepared from DIV 16 days old rat embryos and subjected to transient OGD or oAβ insults as described [15].

### Electrophysiology

LTP measurements were made on 400 μm 3 × Tg mouse slices from 16-month-old male mice using ten theta bursts. Slices were perfused with ODQ (10 μM) and/or NMZ (50 μM) in aCSF perfusate for 30 min before inducing LTP as measured by fEPSP slope measured for 50 min.

### Behavior

The RAWM task has proven informative in the analysis of short-term spatial working memory in AD models and was performed as previously described [58]. NOR and Y-maze were performed as described: preliminary data, tested using a three-way analysis of variance, showed no sex differences in either task for *Aldh2*^*−/−*^ mice, therefore NOR and Y-maze data from male and female mice were combined for analysis [36]. In the NOR, on the test day, animals were allowed to explore the objects until they accumulated a total of 30 s of exploration time, and the discrimination index (difference in time exploring the novel and familiar object, divided by total exploration time) determined. In the Y-maze, spontaneous alternation rate was calculated as the total triads containing entries into each of the three arms without repeated entry into a previously visited arm, divided by the total number of arm entries. STPA has been widely used to test long-term working memory and was performed as previously described [20].

### Biomarkers and immunohistochemistry

Brains were separated by hemisphere for immunohistochemistry or ELISA/immunoblot studies. Frozen hemi-brains were homogenized and serially extracted for biomarker measurement. For EFAD mice, proteins were extracted from the hippocampus using a three step serial extraction as described [38]. Levels of oAβ (Biosensis) Aβ42 (Invitrogen), TNF-α (Invitrogen), pCREB (Biosource), and BDNF (Promega) were determined for soluble and insoluble fractions using sandwich ELISA kits and reported as weight/unit per milligram of soluble protein. Samples for immunoblot analysis of Aβ were incubated in Laemmli buffer containing 6 M urea for 30 min prior to electrophoresis. The following antibodies were used: mouse monoclonal to Aβ (W0-2, Millipore, recognizes residues 4–10 of Aβ), rabbit polyclonal antibody to 4-hydroxynonenal (HNE11-S antibody, Alpha Diagnostics International), mouse monoclonal antibody to PHF-tau (AT8 antibody, Pierce Thermo Scientific), rabbit polyclonal antibody to caspase-3 (ab90437, Abcam), rabbit polyclonal antibody to caspase-6 (ab52295, Abcam), mouse monoclonal antibody to PSD-95 (Pierce Thermo Scientific), mouse monoclonal antibody to total tau (ab64193, Abcam), rabbit polyclonal antibody to phospho-CREB (Ser 133, 06–519, Upstate Biotechnology), rabbit polyclonal antibody to total CREB (06–863, Millipore), mouse monoclonal antibody to β-actin (Sigma). Serial coronal brain sections (30 μm) of paraformaldehyde-fixed hemi-brains were cut frozen using a Leica cryostat in six adjacent series and immunohistochemically processed using antibodies directed against Aβ/APP, a phospho-specific (Ser202/Thr205) tau antibody, and microglia marker Iba1 (primary, 6E10, Covance, 1:2000; AT8, ThermoFisher, 1:1000; anti-Iba-1, Wako, 1:1000; secondary goat anti-mouse IgG for 6E10/AT8, 1:200; goat anti-mouse IgM for anti-Iba-1, 1:200). Quantitative Thioflavine-S staining was performed on frozen hemi-brain 20 μm serial slides. Aβ deposition stereology in cortex and hippocampus were analyzed using quantitative Metamorph software. Brain slices, were incubated with carbachol or vehicle (Basal) for 30 mins and snap frozen. Immunoblot analysis for pCREB was performed using 30 μg protein of hippocampal homogenate, and immunoreactive bands were quantitated by densitometry. Hippocampal slices from WT and *Aldh2*^*−/−*^ mice that had been treated with NMZ or vehicle control for 12 weeks, were incubated with carbachol (50 μM) or vehicle for 30 min and snap frozen, before immunoblot analysis of pCREB in homogenates.

### Statistical analysis

Unless otherwise stated, all data are expressed as mean ± SD or SEM and were analyzed by one-, two- or three-way analysis of variance with either Bonferroni’s or Tukey’s post-hoc test, and/or a Student’s *t* test for unpaired data. A *p*-value of 0.05 or less was considered statistically significant. All statistical analysis was done of GraphPad Prism.

### Ethical committees

The Office of Animal Care and Institutional Biosafety (OACIB) UIC; The Institutional animal care and use committee (IACUC) Colombia University; The University Animal Care Committee (UACC) Queen’s University.

## Abbreviations

AD: Alzheimer’s disease
ALDH2: aldehyde dehydrogenase 2
APOE: apolipoprotein
Aβ: amyloid-beta
BDNF: brain-derived neurotrophic factor
cGMP: cyclic guanosine monophosphate
CMZ: chlormethiazole (also clomethiazole)
CREB: cAMP response element-binding protein
DIV: days in vitro
fEPSP: field excitatory postsynaptic potential
GABA: gamma-aminobutyric acid
HNE: 4-hydroxynonenal
LTP: long-term potentiation
MCI: mild cognitive impairment
NFT: neurofibrillary tangles
NMZ: 4-methyl-5-(2-(nitrooxy)ethyl)thiazol-3-ium chloride
NO: nitric oxide
NOR: novel object recognition
oAβ: oligomeric amyloid-β
ODQ: 1H-[1,2,4]oxadiazolo[4,3-a]quinoxalin-1-one
OGD: oxygen-glucose deprivation
pCREB: phosphorylated cAMP response element-binding protein
PSD-95: postsynaptic density protein 95
RAWM: radial arm water maze
sGC: soluble guanylyl cyclase
STPA: step-through passive avoidance
TBS: theta burst stimulation
TNF-α: tumor necrosis factor alpha
