# Erratum to: A multifunctional therapeutic approach to disease modification in multiple familial mouse models and a novel sporadic model of Alzheimer’s disease

**DOI:** 10.1186/s13024-016-0104-5

**Published:** 2016-05-18

**Authors:** Jia Luo, Sue H. Lee, Lawren VandeVrede, Zhihui Qin, Manel Ben Aissa, John Larson, Andrew F. Teich, Ottavio Arancio, Yohan D’Souza, Ahmed Elharram, Kevin Koster, Leon M. Tai, Mary Jo LaDu, Brian M. Bennett, Gregory R. J. Thatcher

**Affiliations:** Department of Medicinal Chemistry and Pharmacognosy, College of Pharmacy, University of Illinois at Chicago, Chicago, IL USA; Department of Psychiatry, Neuropsychiatric Institute, University of Illinois at Chicago, Chicago, IL USA; Department of Pathology, The Taub Institute for Research on Alzheimer’s Disease and the Aging Brain, Columbia University, New York, NY USA; Department of Biomedical & Molecular Sciences, Faculty of Health Sciences, Queen’s University, Kingston, ON Canada; Department of Anatomy and Cell Biology, College of Medicine, University of Illinois at Chicago, Chicago, IL USA

Unfortunately, after publication of this article, it was noticed that Fig. 6 (Fig. 1 here) was incorrect. The corrected figure can be seen below.

**Fig. 1:**
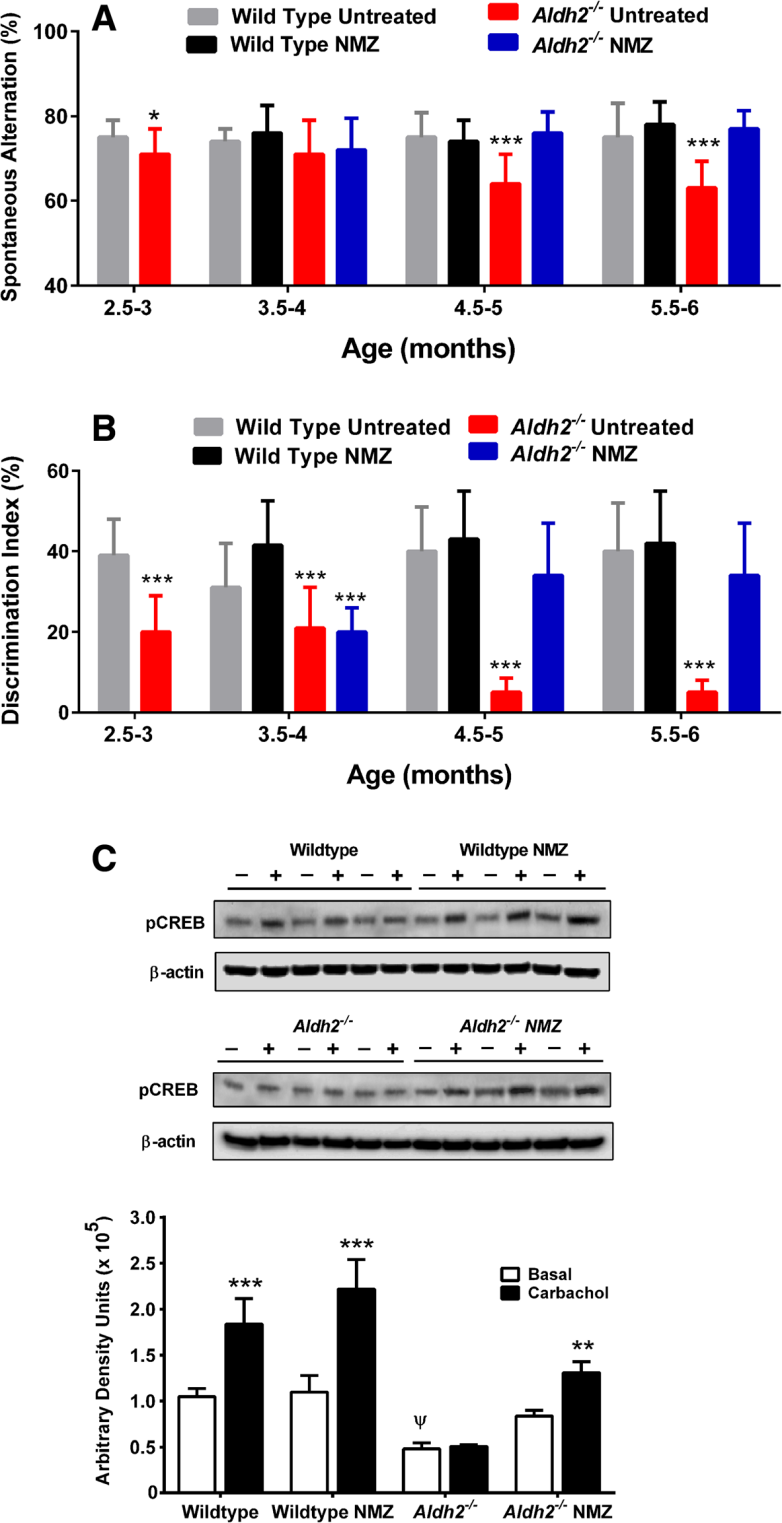
NMZ-treated Aldh2^−/−^ mice show rescued learning, memory and CREB responsiveness in carbachol treated hippocampal slices. Reversal of the age-dependent decline in the spontaneous alternation rate and discrimination index in the Y-maze task (**a**) and NOR task (**b**), respectively, was observed in male and female *Aldh2* ^*−/−*^ mice: after obtaining baseline measurements at 2.5–3 months, mice were randomized to drug or vehicle control groups (*n* = 8–11) and treated with NMZ (20/mg/kg/day p.o.) or vehicle at 3 months of age for a period of 12 weeks. Pre-randomization data were compared by an unpaired *t*-test and post-randomization groups by a one-way ANOVA with a Bonferroni post-hoc test. Hippocampal slices from 6 month old wild type and *Aldh2* ^*−/−*^ mice that had been treated with NMZ or vehicle control for 12 weeks, were incubated with 50 μM carbachol or vehicle (Basal) for 30 mins and snap frozen. Immunoblot analysis for pCREB was performed using 30 μg protein of hippocampal homogenate, and immunoreactive bands were quantitated by densitometry (**c**). Data are presented as the mean ± S.D. (*n* = 3) and were analyzed by a one-way ANOVA with a Bonferroni post-hoc test: * significant differences from basal (** *p < 0.01,* ***p < 0.001); ψ significant difference compared to basal in all other groups (*p* < 0.05)
